# The Association of the Oral Microbiota with Cognitive Functioning in Adolescence

**DOI:** 10.3390/genes15101263

**Published:** 2024-09-27

**Authors:** Oxana Y. Naumova, Pavel V. Dobrynin, Galina V. Khafizova, Elena L. Grigorenko

**Affiliations:** 1 Department of Psychology, University of Houston, Houston, TX 77204, USA; oksana.yu.naumova@gmail.com (O.Y.N.); pavel.dobrynin@times.uh.edu (P.V.D.); galina.khafizova@times.uh.edu (G.V.K.); 2 Vavilov Institute of General Genetics RAS, Moscow 119991, Russia; 3 Department of Molecular and Human Genetics, Baylor College of Medicine, Houston, TX 77030, USA

**Keywords:** youth, cognitive function, saliva, metagenome, oral microbiome

## Abstract

**Background**: A growing body of research supports the role of the microbial communities residing in the digestive system in the host’s cognitive functioning. Most of these studies have been focused on the gut microbiome and its association with clinical phenotypes in middle-aged and older adults. There is an insufficiency of population-based research exploring the association of normative cognitive functioning with the microbiome particularly with the oral microbiota. **Methods**: In this study, using metagenomics and metabolomics, we characterized the salivary microbiome diversity in a sample of 51 males of Hispanic and African American origin aged 12–18 years and explored the associations between the microbiome and the youths’ cognitive performance captured with the Kaufman Assessment Battery for Children II (KABC-II). **Results:** Several bacterial species of the oral microbiota and related metabolic pathways were associated with cognitive function. In particular, we found negative associations between indicators of general intelligence and the relative abundance of *Bacteroidetes* and *Lachnospiraceae* and positive associations with *Bifidobacteriaceae* and *Prevotella histicola* sp. Among metabolic pathways, the super pathways related to bacterial cell division and GABA metabolism were linked to cognitive function. **Conclusions:** The results of our work are consistent with the literature reporting on the association between microbiota and cognitive function and support further population work to elucidate the potential for a healthy oral microbiome to improve cognitive health.

## 1. Introduction

A collective body of research focused on the communication between gut bacteria and neurologic function has strongly supported the concept of the “microbiota-gut-brain axis” (MGBA) [1]. MGBA is underlaid by the interplay between the nervous, endocrine, and immune systems and involves neuronal modulation, immune response, and hormone release [2,3,4]. It has been shown that the MGBA can be implicated in various neurodevelopmental and neurodegenerative disorders [5,6,7,8,9], stress-related responses [10], and neuropsychiatric disorders [11,12,13,14,15,16]. Mechanisms have not been fully established, but several studies have pointed out a special role of microbiota-generated short-chain fatty acids for the microbiota–brain communication pathways [17,18,19].

Although the literature is dominated by the focus on the gut microbiome, recent studies have linked the composition of the oral microbiota (another large digestive system-associated microbial community) with neuropsychiatric disorders [20,21,22,23], the initiation of schizophrenia [24], and cognitive declines related to dementia and Alzheimer’s disease (AD) [25,26,27,28,29]. Collectively, medical, dental, and periodontal research has uncovered the importance of the oral microbiome in systemic disorders, including neuropsychiatric ones, providing empirical evidence of the existence of an oral microbiota–brain axis (OMBA) [20,30]. However, little is known about the theoretical foundation of the OMBA, particularly what mechanisms and pathways the oral microbiota use to engage the central nervous system (CNS). In a recent review [20] summarizing data from animal and human research, four potential mechanisms were highlighted that might underpin the interactions between the oral microbiome and CNS: (1) microbial and metabolite escape through the bloodstream followed by blood–brain barrier disruption; (2) neuroinflammatory response, e.g., expression of proinflammatory cytokines promoted by oral bacteria and their products within the bloodstream and/or CNS; (3) CNS signaling or the presence of neurotransmitter-associated pathways in the oral microbiota, such as tyrosine metabolism and glutamate–glutamine production; (4) response to the host’s neurohormones, e.g., cortisol can modulate bacterial gene expression that may lead to the upregulation of virulence factors in certain bacterial species.

Thus, growing evidence substantiates the association between cognitive functioning and the microbial communities of the digestive tract. Most of this evidence has originated from the gut microbiota case–control studies of clinical phenotypes, e.g., cognitive impairments and AD. Several community-based studies focused on the associations between gut microbial features and normative cognitive functioning have been conducted in middle-aged and older adults from the US [31,32,33,34], Australia [35], and the UK [36], and in school-age children from Israel’s Arab population [37]. Importantly, the data on the potential impacts of the oral microbiota on normal cognitive function collected from population-based samples are scarce. To our knowledge, there is only one recent population-based study of the association between oral microbial composition and executive function and memory changes in a nationally representative population sample of US older adults [38]. This study showed that individuals with higher α-diversity of the oral microbiome had better cognitive performance and were less likely to experience subjective memory changes. It was suggested that systemic inflammation may be a potential mechanism underlying the association between oral microbial dysbiosis (typically linked to periodontal disease) and cognitive function impairment in adults.

Thus, our study is an attempt to contribute to the literature in the field. In this brief report, we present the results of a small-scale study of the association of the salivary microbiome composition with cognitive functioning, in particular with general intelligence, in a community-based sample of ethnic minority youths of African American and Hispanic ancestry aged 12–18 years. The primary hypothesis is that the abundance of distinct bacterial taxa and/or particular bacterial subcommunities of the oral microbiota is significant, although perhaps only mildly or moderately associated with general cognitive functioning in youths.

## 2. Materials and Methods

### 2.1. Sample

The sample included children and youths participating in an ongoing project (NIHCD P20HD091005) focused on the presentation, course, and remediation of severe learning disabilities in the US minority youth involved with the Juvenile Justice System. The inclusion criteria for the participants were ages between 10 and 18; no vision or hearing problems; no diagnosed developmental or intellectual disability; no genetic syndrome or neurological disorder; and “low” levels of trauma, as reported on the Massachusetts Youth Screening Instrument (MAYSI-2) [39]. A total of 5 females and 51 males met the inclusion criteria and provided their saliva samples for the analysis. We excluded females from this study due to the known sex-related variability of the oral microbiota, which is particularly profound in children and adolescents compared to other age groups [40]. Thus, 51 males aged 12 to 18 years (Mean = 15.14 ± 1.37; Median = 15.00) were included in this study; 23 were African American, and 28 were Hispanic. There were no significant differences in the participants’ age distributions between the ethnic groups (Mean = 15.04 ± 1.22 and Mean = 15.18 ± 1.42 for African American and Hispanic youth, respectively).

### 2.2. Cognitive Assessment

Each individual’s cognitive ability and mental processing were assessed using the Kaufman Assessment Battery for Children II (KABC-II) [41]. The KABC-II has 18 subtests grouped into four scales in accordance with Luria’s interpretive model: sequential processing, simultaneous processing, learning ability, and planning ability, and yields the general intelligence composite score—the Mental Processing Index (MPI). MPI scores were utilized to analyze the association analysis with the microbiome. There was a broad range of variability in the participants’ cognitive functioning: MPI Min = 60, Max = 110, Mean = 84.27, SD = 10.43, Median = 84.00, and Mode = 84.00. The Shapiro–Wilk test did not show evidence of non-normality in the MPI scores’ distribution (W = 0.99, *p* = 0.98). The Kruskal–Wallis tests revealed no significant differences in cognitive performance between the racial/ethnic groups. Individual data on the participants’ demographics and KABC-II scores are presented in Supplementary Table S1.

### 2.3. Microbiome Profiling

Saliva specimens were collected using the OG-500 Oragene-DNA collection kit (DNAGenotek, Ottawa, ON, Canada), which contains a preservative that stabilizes the salivary DNA content, including those originating from the oral microbiota [42]. Salivary DNA was isolated using prepIT.L2P reagent as per the manufacturer’s protocol for DNA extraction and purification (DNAGenotek). DNA sequencing was performed at the Human Genome Sequencing Center of Baylor College of Medicine, using the Illumina standard PCR-free protocol for library preparation and the Illumina sequencing platform, with a target sequencing depth of 30× human genome coverage. Raw sequencing reads were mapped to the GRCh38 human genome using the Burrows–Wheeler Aligner (BWA) (https://github.com/lh3/bwa) [43], followed by processing the resulting alignments with SAMtools v. 1.18 (https://github.com/samtools; accessed on 1 September 2023) [44]. Sequencing reads unmapped to the human genome were used for the taxonomic profiling of the metagenome with the MetaPhlAn2 pipeline (https://github.com/biobakery/MetaPhlAn; accessed on 1 January 2024) [45,46]. The functional profiling of the metagenome was performed using the HMP Unified Metabolic Analysis Network (HUMAnN 3.0) (https://github.com/biobakery/HUMAnN; accessed on 1 January 2024) [46].

### 2.4. Statistical Analysis

The primary analysis was performed at the family and species taxonomic levels. Indices of α-diversity were derived from the relative abundance metrics and estimated as richness (the number of distinct taxa per an individual’s microbiome) and the Shannon–Weaver diversity index (*H*), a function of both richness and evenness [47]. Intergroup analysis of the microbiome diversity and composition indices was performed using the Wilcoxon–Mann–Whitney test (WMW). An association analysis of the cognitive score (MPI) with the microbiome diversity and composition indices and the related microbiota metabolic pathway abundance was conducted using Spearman’s rank-order correlations (R_S_) [48]. The Benjamini–Hochberg adjusted *p*-values (*q*) were obtained whenever appropriate to control for multiple testing. Prior to the correlation analysis, first, the taxa with a count of zero in at least 25% of individual saliva samples were removed to minimize the influence of rare taxa and spurious results. Second, the indices of relative abundance were adjusted for potential confounding effects of the participants’ age and ethnicity; the standardized residuals were used in further analyses. Both factors are known to be associated with oral microbiome composition and diversity. In particular, aging has been associated with lower within-sample and higher between-sample diversity in the oral microbiota [40], and ethnic group-specific patterns of the microbiome composition have been registered across various age groups [49,50,51].

## 3. Results

### 3.1. Microbiome Composition and α-Diversity: Functional Profiling of the Metagenome

A total of 470 bacterial species from 92 families were identified in 51 individual saliva specimens; the data on the relative abundances of these taxa are presented in Tables S2 and S3 for species and families, respectively. The number of taxa within an individual microbiome significantly varied: from 176 to 327 and from 45 to 72 for species and families, respectively. The richness and Shannon *H* indices indicated high individual diversity of the microbiome composition and inter-individual variability in the level of α-diversity: for species, Mean richness = 258 ± 37 and Mean *H* = 8.9 ± 1.8, and for families, Mean richness = 60 ± 5 and Mean *H* = 3.9 ± 0.3 (Table 1 and Table S1). However, 59 species and 33 families were detected across all 51 individuals. Descriptive statistics of the relative abundance of the 12 most common bacterial families representing over 80% of the total relative abundance in the sample are shown in Table 2. In accordance with published data [52,53], *Streptococcus*, *Neisseria*, and *Prevotella* were identified as the core members of the human salivary microbiome.

Based on the metagenome functional profiling, a total of 412 metabolic pathways were identified. Of those, 290 were detected in all individual salivary samples. The distribution of the pathways’ relative abundances across individuals is presented in Table S4. The most common and abundant were pathways related to peptidoglycan (major structural polymer in most bacterial cell walls) biosynthesis and maturation and to de novo nucleotide biosynthesis that is essential for DNA replication, cellular signaling, and energy metabolism and strongly linked with the virulence of bacterial pathogens [54].

### 3.2. Microbiome Features and Participants’ Demographics

After the correction for multiple comparisons, there was no significant association of the participants’ age with α-diversity and the distribution of distinct phyla and metabolic pathways. However, using an FDR of <0.20, we observed a significant negative correlation between the participant’s age and the abundance of several bacterial families: *Aerococcaceae* (r = −0.4568, *p* = 0.001), *Ca. Nanogingivalaceae* (r = −0.4144, *p* = 0.003), *Promicromonosporaceae* (r = −0.4007, *p* = 0.004), and *Streptococcus gordonii* sp. (r = −0.4856, *p* = 0.0003). This *Streptococcus* species is an initial colonizer of the periodontal environment, which initiates dental plaque formation [55].

Although the literature reports on the ethnic variation in the composition of the human oral microbiome [50,51], we did not observe a significant difference between ethnic groups after *p*-value correction for multiple testing. However, we registered nominally significant (at an unadjusted *p* < 0.05) interethnic variability in the abundance of seven metabolic pathways, ten species, and four microbial families: *Peptostreptococcaceae*, *Aerococcaceae*, *Bifidobacteriaceae*, and *Leptotrichiaceae* (Table S5). In addition, for the *Leptotrichiaceae* phylum (Abundance Mean = 0.0078 ± 0.0060), the difference in abundance remained significant at the FDR threshold of 0.20: t(df) = 3.26(49), *p* = 0.002; WMW *Z* = 3.23, *p* = 0.0012. We attribute this lack of significance to the combined effect of a small sample size and the shared living environment of the participants, who had stayed at the HCJPD facility for six months or longer.

### 3.3. Association between Oral Microbiome and General Intelligence

With regard to α-diversity, both richness and Shannon H did not show a significant (at a suggestive *p*-value of <0.05) association with general intelligence in adolescents. After removing taxa and metabolic pathways with a count of zero in at least 25% of participants, 54 families, 192 species, and 314 pathways (Tables S2–S4) were included in the correlation analysis. Accounting for multiple comparisons, a number of microbiome features showed a weak but significant (at a q < 0.10) correlation with general intelligence captured by the KABC-II MPI score (Table 3). In particular, we found 11 species, four bacterial families, and two metabolic pathways associated with the MPI. The majority of correlations were negative: the abundance of 10 species and three bacterial families (*Flavobacteriaceae*, *Cardiobacteriaceae*, and *Candidatus Gracilibacteria*). Among metabolic pathways, peptidoglycan biosynthesis V and 4-aminobutanoate degradation III had a negative correlation with the MPI score. Yet, the *Bifidobacteriaceae* family and *P. histicola* sp. had a positive correlation with the MPI: R_S_ = 0.3216 and 0.3598, respectively (Table 3). Thus, despite this study’s small sample size, these results support our initial hypothesis, suggesting that the abundance of distinct bacterial taxa is associated with variation in cognitive functioning in healthy samples.

## 4. Discussion

In this community-based sample, we explored the link between the oral microbial community composition and the general intelligence scores assessed with a widely used instrument, the KABC-II, in a cohort of Black and Hispanic US adolescents. Within-person microbial diversity was not associated with cognitive functioning; this contradicts the findings of a population-based study of US older adults, where participants with higher oral microbial α-diversity demonstrated better cognitive performance [38]. Of note is that studies linking the gut microbiota α-diversity with cognitive performance in healthy adults have reported contradicting results on both the presence [34,36,37] and the absence of the association between microbiota diversity and cognition [31,35,56]. It is possible that this relationship is more complicated and depends on the participant’s demographics, health status, lifestyle, and other confounding factors; further research is needed to clarify the presence and strength of this association.

The oral microbiota is the second largest microbial community after the gut community; it harbors over 700 species of bacteria and plays a key role in immunological and physiological functions [53,57]. Recent studies have highlighted the oral–gut microbial transmission of the taxa active along the gastrointestinal tract that, in turn, can affect immunity and promote/regulate pathogenesis [58,59,60]. In our sample, we identified, on average, about 250 distinct species and 60 bacterial families within an individual microbiome. We found an association between general intelligence indices and the abundance of several distinct bacterial species and families, with a significant predominance of negative correlations. There were several associations consistent with the findings that have been provided by empirical studies exploring microbiome–cognitive functioning relationships. In particular, in our sample, the MPI score decreased with an increase in the abundance of *Cardiobacteriaceae*, *Candidatus Gracilibacteria*, and *Flavobacteriaceae*. The last is the largest family in the phylum *Bacteroidetes*. An elevated abundance of the *Bacteroidetes* phylum in the gut microbiota has been associated with lower executive function [61] and has been observed in cognitive impairment groups, e.g., older adults with poor cognitive functioning in late life [62], and in patients with insomnia [63], Mild Cognitive Impairment (MCI) [64,65], AD, and dementia [66,67]. Similar to the gut microbiome, a higher abundance of *Bacteroidetes* was registered in the subgingival microbiome of older adults with cognitive decline [26].

At the species level, we found a negative association with the MPI scores for representatives of the *Bacteroidetes* phylum such as *P. conceptionensis*, *Capnocytophaga SGB2480,* and *C. sputigena.* In addition, two species from the family *Lachnospiraceae* (*Lachnospiraceae* oral taxon 500 and *Oribacterium* sp oral taxon 078) were negatively related to the intelligence scores in our sample. Similarly, it has been shown that the increased presence of *Lachnospiraceae* family members in the subgingival microbiota increased with declining cognition in older adults [26]. In the gut microbiome, a higher abundance of *Lachnospiraceae* has been associated with more errors in spatial working memory tasks in older adults [63] and lower communication skills in toddlers [68].

Conversely, the abundance of the members of the *Bifidobacteriaceae* family and the *P. histicola* species was positively associated with cognitive functioning; this finding also has support in the relevant literature. Thus, in the gut microbiome, an increased presence of the *Bifidobacterium* genus has been negatively associated with cognitive function in late life and has been registered in participants with varying cognitive and depressive symptoms [62,69], AD and dementia [66,70,71], and deteriorated Parkinson’s disease [72]. In addition, it has been shown that bifidobacterium-based probiotic interventions might be promising instruments to improve cognition [73]. Here, we provide one of the first pieces of evidence of a positive association between the abundance of *Bifidobacteriaceae* in the oral microbial community and cognitive functioning.

Along with *Bifidobacteria*, *P. histicola* is another human gut commensal that has shown probiotic efficacy against arthritis [74], estrogen deficiency-induced depression [75], and Multiple Sclerosis (MS) [76]. Several studies have reported on the association between a lower abundance of *P. histicola* in the gut microbiota and cognitive impairments related to neurological lesions. Among those are MS [77,78] and vascular dementia (VaD) [79], whereas *P. histicola* abundance is increased in response to disease-modifying therapy in MS patients [76], and *P. histicola* transplantation improves cognitive functioning in VaD rats [79].

There is a proof of concept in the literature that the gut microbiome influences cognitive function through the MGBA, which engages endocrine, immunological, and neuroactive pathways to communicate with the CNS and is mediated by microbial neurotransmitters (e.g., GABA and catecholamines), metabolites (e.g., short-chain fatty acids, tryptophan, and nucleotides), and signaling molecules stimulating the release of peptides and hormones from enteroendocrine cells [80,81,82,83,84,85]. Empirical research has provided evidence that glycogen biosynthesis, formaldehyde oxidation, and petroselinate biosynthesis were especially enriched in the gut microbiome of individuals with impaired cognition [82]. A recent study of functional network connectivity showed that gut *Bacteroides* are linked to multiple metabolic pathways (primarily, those related to arginine, phenylalanine metabolism, and the biosynthesis of unsaturated fatty acids) associated with functional network connectivity, which, in turn, can mediate the well-established associations between Bacteroides abundance and cognition [81].

Little is known about the oral microbiota metabolites and pathways related to cognitive functioning. The main findings originate from studies focused on the gut microbiota. In addition, the oral and gut microbiotas differ in terms of their resident bacteria; consequently, their metabolomics profiles also differ [86]. However, like the analysis based on taxonomic profiling, our results of the functional annotation of the salivary microbiome, followed by the association analysis with cognition, seem to be consistent with the findings of the literature. We identified two metabolic pathways—peptidoglycan biosynthesis V and 4-aminobutanoate degradation III—and both had negative correlations with general intelligence. The first is a general non-specific superpathway related to peptidoglycan synthesis, which is vital for the majority of bacterial species and essential for the expansion of the scaffold during cell elongation and the formation of a septum during cell division. This finding may indicate the potential negative association between cognitive functioning and the overall bacterial abundance in the oral cavity and, in turn, oral health. However, the second pathway is related to the metabolism of 4-aminobutanoate (GABA)—the major inhibitory neurotransmitter in the mammalian brain, which can be synthesized by many bacterial strains and is considered a key neurotransmitter mediating the microbiome–brain communication pathway of the OMBA.

The main limitation of our study is the sample size; it is relatively small for the analysis of multivariate and high-dimensional data such as microbiomic and metagenomic ones. This significantly limits our choice of analytical methods and models. Despite this limitation, the results of this study support an association between the oral microbiome and general intelligence and contribute to the sparse literature on the potential relationship of the oral microbiota with cognitive functioning in youths.

## 5. Conclusions

This study explored the associations between the salivary microbiome and general cognitive performance in a US multiethnic community-based sample of male adolescents. We found negative associations between the indicators of general intelligence and the relative abundance of *Bacteroidetes* and *Lachnospiraceae* in the oral microbiota. At the same time, the abundances of *Bifidobacteriaceae* and *Prevotella cisticola* spp. were positively associated with cognitive performance. These results indicate that interventions aimed at “a healthy oral microbiome” may be a promising avenue for enhancing cognition. In addition, the analysis of the microbiota-related metabolic pathways revealed that pathways related to GABA metabolism may be among the mechanisms underlying the association between microbiota and cognitive performance as a function of the CNS. These results provide a shred of empirical evidence supporting the concept of the “microbiota–brain axis”—a system of communication between the microbiota and the brain.

## Figures and Tables

**Table 1:** The number of bacterial taxa detected in the salivary specimens from 51 participants and the indices of α-diversity: the richness or number of taxa and Shannon *H*.

	Family	Species
Omnipresent	92	470
Present in 100% of samples	33	59
Present in 75% of samples	54	192
Present in 50% of samples	58	271
Richness, range	45–72	176–327
Richness, Mean (SD)	60 (5)	258 (37)
Shannon *H*, range	3.3–4.5	4.7–12.8
Shannon *H*, Mean (SD)	3.9 (0.3)	8.9 (1.8)

**Table 2:** Descriptive statistics of the relative abundance of the 12 most common families that make up about 80% of the total bacterial abundance in saliva.

	Min	Max	Mean	SD	Median
*Prevotellaceae*	0.0385	0.2729	0.1291	0.0590	0.1202
*Neisseriaceae*	0.0068	0.4028	0.1290	0.0939	0.1222
*Streptococcaceae*	0.0369	0.2200	0.0908	0.0426	0.0782
*Veillonellaceae*	0.0327	0.2931	0.0893	0.0524	0.0823
*Pasteurellaceae*	0.0235	0.3021	0.1150	0.0669	0.0968
*Actinomycetaceae*	0.0050	0.1393	0.0437	0.0322	0.0351
*Micrococcaceae*	0.0057	0.1345	0.0429	0.0293	0.0338
*Porphyromonadaceae*	0.0032	0.1237	0.0400	0.0283	0.0349
*Ca. Saccharibacteria*	0.0020	0.1221	0.0397	0.0291	0.0312
*Fusobacteriaceae*	0.0068	0.0815	0.0372	0.0176	0.0355
*Bacillales*	0.0056	0.0785	0.0273	0.0194	0.0236
*Burkholderiaceae*	0.0008	0.1347	0.0242	0.0314	0.0100

**Table 3:** The oral microbiome taxa and related metabolic pathways showed a significant (at a *q* < 0.10) correlation with general intelligence in adolescents captured by the KABC-II MPI score.

Microbiome Feature		R_S_	t	p	q
Species	*P. histicola*	0.3598	2.70	0.0033	0.0625
	*Prevotella conceptionensis*	−0.3580	−2.68	0.0049	0.0678
	*Streptococcus cristatus*	−0.3919	−2.98	0.0022	0.0625
	*Capnocytophaga sputigena*	−0.3919	−2.98	0.0022	0.0625
	*Capnocytophaga* SGB2480	−0.3670	−2.76	0.0040	0.0625
	*Campylobacter* SGB19317	−0.3841	−2.91	0.0027	0.0625
	*Actinobaculum* sp oral taxon 183	−0.3675	−2.77	0.0040	0.0625
	*Lachnospiraceae* oral taxon 500	−0.4304	−3.34	0.0008	0.0625
	*Oribacterium* sp oral taxon 078	−0.3649	−2.74	0.0042	0.0625
	GGB4733 SGB6557	−0.4247	−3.28	0.0009	0.0625
	GGB3385 SGB4472	−0.4194	−3.23	0.0011	0.0625
Families	*Bifidobacteriaceae*	0.3216	2.75	0.0033	0.0728
	*Flavobacteriaceae*	−0.3784	−2.86	0.0031	0.0728
	*Cardiobacteriaceae*	−0.3543	−2.65	0.0054	0.0766
	*Candidatus Gracilibacteria*	−0.3692	−2.78	0.0038	0.0728
Pathways	PWY-6470: peptidoglycan biosynthesis V	−0.4594	−3.62	0.0006	0.0904
	PWY-5022: 4-aminobutanoate degradation III	−0.4468	−3.50	0.0009	0.0904

## Data Availability

The data generated in the current study are provided in the Supplementary Tables; the primary sequencing data on the metagenome are available upon reasonable request from the corresponding author.

## Supplementary Materials

The following supporting information can be downloaded at: https://www.mdpi.com/article/10.3390/genes15101263/s1. Table S1: Individual data on the participants’ demographics, cognitive ability, and oral microbiome diversity. Table S2: The relative abundances of 470 microbial species identified in the salivary specimens from 51 individuals; the 192 most common taxa detected in at least 75% of samples are marked in bold. Table S3: Relative abundance of 92 bacterial families identified in salivary specimens from 51 individuals; 192 most common taxa detected in at least 75% of samples are marked in bold. Table S4: Relative abundance of metabolic pathways; most common pathways detected in at least 75% of samples are marked in bold. Table S5: The intergroup comparison of the relative abundances of the bacterial taxa and metabolic pathways with the Wilcoxon–Mann–Whitney test.
